# The Eureka Moment: An Interview with Sir Alec Jeffreys

**DOI:** 10.1371/journal.pgen.1000765

**Published:** 2009-12-11

**Authors:** Jane Gitschier

**Affiliations:** Department of Medicine and Pediatrics, University of California San Francisco, San Francisco, California, United States of America

In 1984, while tracking the veins of globin gene evolution and panning the human genome for hypervariable linkage markers, Sir Alec Jeffreys accidentally struck gold—he discovered a way to identify any human being by a DNA “fingerprint”. To use Jeffreys' words, he has been “branded” by DNA fingerprinting, but he delights in its application and the hook it provides for public curiosity about science. Like Jeffreys himself, I wanted to dig below the surface of this discovery as well as that of another genetic nugget—the intervening sequence—found as a post-doctoral fellow seven years earlier.

On the heels of my interview with Adrian Bird (published in the October issue of *PLoS Genetics*), I made my way to Jeffreys through another branch of the British Rail system. When I arrived at his building on the leafy Leicester campus about 45 minutes early for our appointment, his assistant suggested I get a cup of coffee while Jeffreys finished his experiment. I certainly wouldn't have needed one. Jeffreys (Image 1) is an animated speaker, with a resonant voice and a rapid delivery of succinct clauses strung together in run-on sentences. His story could have cut through anyone's jet lag.

**Figure:**
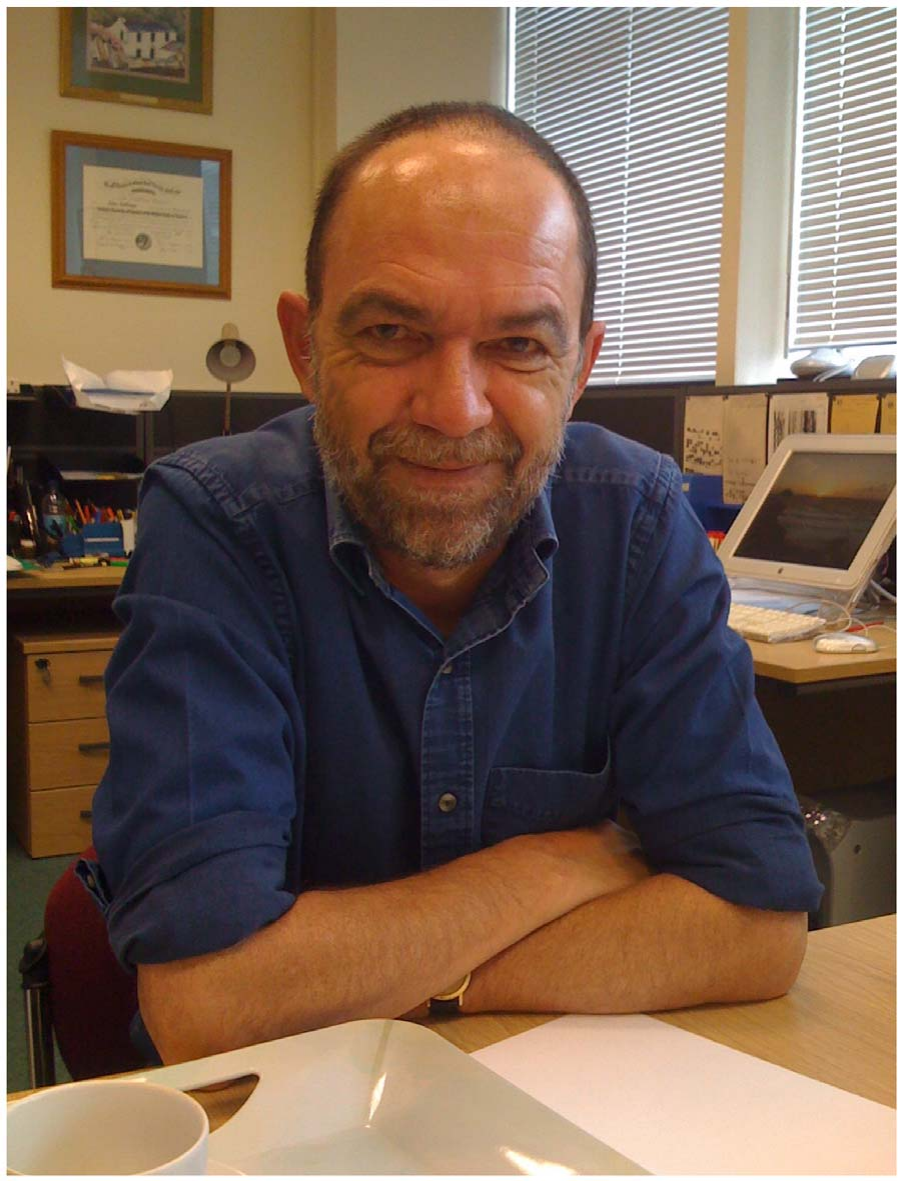
Sir Alec Jeffreys.

**Gitschier:** I didn't realize that you still work in the laboratory.

**Jeffreys:** I certainly do!

**Gitschier:** Tell me about the experiment you were just doing.

**Jeffreys:** Right, well, we won't go into the gory details. Copy number variation [CNV] in the human genome is a real hot topic at the moment.

**Gitschier:** The kind of variations people are looking for in association with autism and psychiatric diseases.

**Jeffreys:** That's exactly right. It's a common phenomenon, and we've actually known that for decades. What we're doing is going back to some of the absolutely classic examples of CNV. These are in my favorite gene family—the globin genes—and that's where I cut my scientific teeth.

**Gitschier:** We're going to be coming back to that!

**Jeffreys:** Right. So, what I've done in my scientific career is this gigantic circle, starting off in globin genes, going all around the place in forensics, and returning back to my first love. The experiment I'm doing at the moment is looking at de novo copy number variation in the fetal γ-globin genes at the single molecule level in both somatic and germline DNA.

All of this comes out of my work on recombination hotspots. And the general feeling was that recombination hotspots function at meiosis—they drive allelic recombination, and they may well drive ectopic recombination.

**Gitschier:** Define “ectopic.”

**Jeffreys:** The term ectopic originally came from yeast and it applied there to a situation in which you have a sequence repeated, say here and there, so that they can undergo unequal crossover and cause duplication and deletion. “Ectopic” recombination means it's “out of place.”

As of yesterday, I found there is copy number instability not just in the germline, but in somatic DNA. That largely rules out meiosis and meiotic recombination hotspots. Even in the germline it is quite clear that the substantial proportion, possibly the great majority of rearrangements, are again pre-meiotic, arising during germ cell development. We're trying to drill down below the applied genetics [looking for variation associated with disease], to some of the fundamental mechanisms, to understand the dynamics of rearrangements in the human genome.

So, if you want to put a simple summary on what this lab is about, it's about human DNA diversity and the processes that generate it.

**Gitschier:** OK! Now let's get to the first question on my list, which indeed is about globin. It's about the period of your post-doc in Amsterdam. Why did you go there and why work with Flavell?

**Jeffreys:** OK. I did my D. Phil. at Oxford University on human somatic cell genetics. Then went to a Biochemical Society meeting and chatted with a chap named Piet Borst, a very senior scientist, who at the end said, if you are interested in doing a post-doc with me, just let me know.

And I thought, that's great, ′cause I wanted to get out of Oxford, and Holland I really fancied because the language wasn't going to be a problem; everyone speaks English. So, I got myself an EMBO fellowship to work with Piet on yeast tRNA genes.

In 1975, the door was clearly opening on molecular genetics, before that, it wasn't worth talking about.

**Gitschier:** Expand on that statement.

**Jeffreys:** I remember very clearly. There was a colleague of mine at Oxford called David Finnegan and we're waiting in the lunch queue and he wanted to go off to the States, and I said what's the project, and he said the idea is to take *Drosophila* DNA and to try to stick bits of that into lambda phage.

**Gitschier:** With David Hogness?

**Jeffreys:** Right. And the penny dropped then, that this was going to be the way forward.

I get to Amsterdam, and Piet said, you can work on this if you like, but you might also like to have a chat with this guy Dick Flavell, he's got a collaboration with Charlie Weissman in Zurich, on trying to isolate a mammalian gene. And I thought—whoa! That's sounds really exciting. The idea of the project was to get to a single-copy gene. No one had ever done that in a mammalian system. The only one we could possibly do, we felt, was either rabbit α- or rabbit β-globin, because the mRNA had been purified. The gene isolation would be by physical purification.

**Gitschier:** No cloning?

**Jeffreys:** Well, cloning came in right at the end. It simply wasn't around at the time. It was by hybridization enrichment with prodigious quantities of DNA [from rabbit liver]. The experiment was to cut it up with EcoRI restriction enzyme. Remember, this is back in the days when you couldn't just buy enzymes off the shelf, you had to *make* them.

Then denature the DNA and hybridize it to globin mRNA. This was a two-pronged attack. In Amsterdam we were going to use the mRNA to pull out the complementary strand, heavily enriched, and in Zurich, Charlie Weissman had managed to make a cDNA so he could pull out the other strand, and the idea was to purify our complementary strands and then meet somewhere in the middle to hybridize the two stands back together. Then, because this was an EcoRI fragment, we could then pop it into a vector that we hoped someone was about to develop.

**Gitschier:** How were you selecting the mRNA?

**Jeffreys:** We were selecting by attaching mercury to the RNA and then capturing it on a thiol column.

**Gitschier:** That's a dangerous experiment.

**Jeffreys:** Oh, the whole thing was horrendous. We were using radioactive mercury.

**Gitschier:** But hold on. Since there was no reason to suspect that there were intervening sequences, what is the point of going after the gene?

**Jeffreys:** Nobody had ever seen a mammalian gene. No one had any idea of what it would look like.

**Gitschier:** So, the idea was to get something bigger than the mRNA itself.

**Jeffreys:** Yes, that's right. To look at the flanking regions. Basic academic curiosity.

During that experiment, we had to develop methods for monitoring purification, and the only way we could see to do that was to use Ed Southern's blotting technique, which at that point was only a year or two old.

So, as we purified the DNA we could monitor the fractions just by running them out on an agarose gel, doing the Southern blot and then hybridizing with an appropriate complementary probe. And that not only worked, but we could actually *see* the fragment of DNA we were trying to purify in the starting EcoRI digest of genomic DNA.

**Gitschier:** Hadn't he shown that before?

**Jeffreys:** No, Ed was desperately trying to get this going. I know Ed extremely well, and there was a bit of discomfort on my part thinking that we had trampled on his patch. On the other hand, that is what we needed to do.

Having got the ability to detect down to the single gene level, we thought we should see if we could make a restriction map around the gene, which is what we did.

**Gitschier:** Were there EcoRI sites in the cDNA?

**Jeffreys:** No. The cDNA had been cloned by Tom Maniatis, and we pretty quickly moved over to using his rabbit β-globin cDNA that he very generously provided to act as a probe for monitoring. We just wanted to check that everything was OK. And we built up a restriction map around it [on genomic DNA via Southern blotting].

We then discovered that there was an EcoRI site right smack bang in the middle of the gene! [That meant] our enrichment experiment was a total disaster, because we would have purified one end of the gene in Amsterdam, and in Zurich, they would have purified the other end of the gene, and to put them together, there would be nothing. The flop of the millennium that was!

But, the question then was, what the hell is the EcoRI site doing in the middle of the gene? And then we started to do more and more fine-mapping and it was clear there was a huge gap in the gene.

I remember sitting down with my Dutch technician, saying we've got the restriction mapping data, let's try putting all this together. And I knew it was just nuts, but I thought we could solve it if we just put an extra dollop of DNA inside the gene. All of this was done without reference to Phil Sharp and Rich Roberts's work with adeno [which was happening at the same time]. I knew instinctively that this was something pretty exciting. And then, Dick was over for, I think, a Cold Spring Harbor Meeting, and everything started falling together. About the same time, Phil Leder managed to clone in lambda the mouse β-globin gene and showed by electron microscope analysis that there was additional sequence inside the gene. But the trouble there was it had been cloned in *E. coli* and perhaps it was an insertion sequence. And then [there was] Chambon's ovalbumin gene story.

Looking back on it, basically in 1977, introns were going to be discovered. Full stop. The technology had arrived to the point where the discovery was inevitable. I think all of us in the field were grateful that we just happened to be at the right place at the right time.

When it was time to leave Amsterdam, one possibility was to do a post-doc with Ed Southern up in Edinburgh. He's a great guy and the stuff he was doing was fantastic. He's one of my heroes. We are actually quite similar. We like fiddling around with things. He gave this wonderful quote a few years ago that he misses the days when he could get at the data before the computer did.

But at the same time, I thought I'd like to try running my own lab, and out of the blue came a phone call from this guy called Bob Pritchard who founded this Department [of Genetics] in the early 60s. He said, “Would you be interesting in coming for an interview?” I said, “Where is it?” He said, “Leicester.” And I said, “That will be fine.”

I put the phone down and I said, “Where the hell is Leicester?” All these Dutch people were running around trying to find a map of Europe.

**Gitschier:** Pre-internet.

**Jeffreys:** Pre-everything! These were the days if you wanted a sequence you had to get out a typewriter and type it in.

So, I visited Leicester and I immediately fell in love with the department. I came as a temporary lecturer, and I'm still here 32 years later, so it says something about the environment. I love it here.

So, the question then was, what was I going to do? It was clear that carrying on with the intron work was not going to be viable. Suddenly everybody was moving into the field—evolution of introns, mechanisms of splicing, etc. I thought, take your education in human genetics and your new-fangled molecular biology and stick them together. If you can pick up specific bits of human DNA, then you should be able to scan for variation. Variation that affects a restriction enzyme site will manifest as what is now called, I think very uglily, an RFLP [restriction fragment length polymorphism].

So, that was our first quest. By early 1978 we had picked up our first RFLP, a rare variant in a single individual. Again, these were in the globin gene clusters, because again, these were the only genes for which probes existed at that time. Really excited, but we got pipped to the post because Kan and Dozy published their RFLP and the association with sickle cell disease.

**Gitschier:** I think they just bumped into that discovery.

**Jeffreys:** What we had done was to do a fairly systematic survey for RFLPs in the β-gene cluster.

**Gitschier:** What made you think that there would be variation in restriction sites among people?

**Jeffreys:** I can't remember. It seemed fairly obvious at the time. I knew enough human genetics to know that there must be a significant amount of variation in DNA sequence. I'd been brought up in the days of serology and biochemical genetics, enzyme polymorphisms, and we knew that that was sampling only a tiny proportion of all diversity in the genome. So, if there is diversity, then it will be agnostic with respect to restriction sites, so if you luck out, you'll find a polymorphism that hits a restriction site and that makes it assayable.

Having come up with these RFLPs, we then got fed up with them, cause everyone was doing it. So, we then started thinking that surely in this enormous human genome, there must be bits of DNA that are more variable than these RFLPs, and we thought intuitively that the right place to look was tandem repeat DNA. I've been brought up in the school of satellite DNAs, which was the only class of DNA you could purify going back to the old cesium chloride density gradient days. The satellite DNAs incidentally show a lot of variability in copy number.

I felt intuitively that if you had local tandem repeat sequences on a smaller scale in the genome, they'd have potential variation as well. The hypothesis was that there may be bits of DNA with repeats, maybe 10 or 20 bases long repeated 10 or 20 times, so we started all kinds of crazy experiments trying to physically purify these bits of DNA from the human genome.

Then in 1980 Arlene Wyman and Ray White described the first hypervariable locus, so I thought WOW they do exist! But their interpretation was one of transposition. Why? Because they came from a transposable element background. So, quite reasonably, they were thinking, OK it's hypervariable because we've got a transposable element that is moving in and moving out, taking DNA with it and creating this length variation. But, I read their interpretation of transposition and I just felt not so sure about that. So, we then started redoubling our efforts and still getting nowhere at all.

Then Graeme Bell described the sequence of the human insulin gene and right next door to it was a minisatellite—a highly variable tandem repeat region. And then Doug Higgs in the α-globin region.

**Gitschier:** What approach were you using to try to find these variable minisatellites?

**Jeffreys:** It was primarily physical enrichments. These sequences might have unusually fast reannealing kinetics, so you could do a COT approach. Or, since these sequences might be quite long but consisted of repeats over and over again they would tend to be resistant to restriction enzymes, so, if you took a load of common cutting restriction enzymes, you would leave these things intact.

We were still getting nowhere. But meanwhile [in a separate project], we were doing some globin gene family evolution work. We thought, OK there is a missing gene in the story, and that is myoglobin. Could we get the myoglobin gene out and see how it fitted in to the hemoglobin gene family as a very diverged member of that family?

So, this is really the start of the DNA fingerprint story, because we got the human myoblobin gene and found a minisatellite inside the intron.

**Gitschier:** How did you find that?

**Jeffreys:** By sequencing. It wasn't variable between people, but I realized I had seen this sequence somewhere else. So, I went back and looked at the α-globin and the insulin minisatellites, and you could see this sort of vague suggestion that there might be some sort of shared sequence in there. So, we then took that myoglobin minisatellite and hybridized it to a human lambda library and lo and behold a number of clones lit up. We then started systematically isolating those clones, showed that they contained minisatellites and some of them were pretty variable loci.

**Gitschier:** So, you were checking this on a Southern blot?

**Jeffreys:** Southern blot and characterizing by sequencing. And, as we were building up the repeat sequences from the clones coming out of the library, the shared sequence motif, the minisatellite core, became more and more obvious. It was a short sequence, about 15 bases long, embedded within the repeats of the minisatellites. It was almost as if this was some kind of sequence driving this tandem repetition. But, more important, it could give you a much more effective generic way of getting minisatellites out of the genome, because rather than using this crummy myoglobin probe, you take a probe that consists of just this core sequence repeated over and over again.

So, we took that and hybridized it to a Southern blot, which happened to have [DNA from] the lab technician and her mom and dad. We got this fuzzy splodgy mess, but the DNA fingerprint was absolutely obvious. We got a pattern like a fuzzy bar code. These patterns were individual specific, and seemed to be inherited within the family. That was a real eureka moment, because we were suddenly onto something completely new, which was DNA-based identification.

Recall, the driver for this experiment was medical genetics. You needed these improved markers for facilitating construction of linkage maps of the human genome and helping in linkage analysis of inherited disease. This thing would have been useful were it just a single location in the genome, but the fact that there were multiple copies of the repeat sequence in the genome gave it a new meaning, in terms of DNA identification.

When I talked about it in a Department seminar, and then speculated about what we could use this for, like catching rapists from semen—about a third of the audience fell over laughing. It sounds bizarre now because it's so blindingly obvious that you can use DNA for this, but believe me, back in the 80s it was simply not there. The only reason I came up with the idea of DNA-based identification was that it just hit you in the face!

So, within the first day, we saw identification, we could foresee forensic analysis if DNA survived in forensic specimens, zygosity testing in twins, paternity testing, and immigration disputes. Just like drawing up a shopping list—if we could get this technology improved, what it could be applied to.

**Gitschier**: I clearly remember that Nature paper [1985] involving the immigration dispute that you helped to settle, the case where a boy was threatened with deportation because the immigration authorities alleged he wasn't the biological son.

**Jeffreys:** That was the first DNA case tackled anywhere in the world, and it is still my favorite case because I was there at the tribunal where they dropped the case against the boy, when the mother was told—and just the look in that mother's eyes! She had been fighting the case for two years.

That was my golden moment. Without DNA, he could have been deported.

**Gitschier:** That set of events must have built up momentum for you and your lab.

**Jeffreys:** Oh, it did. We hadn't realized how many thousands of *other* people were trapped in these disputes! So, the next thing was a complete avalanche of letters and phone calls; people were turning up at my home!

**Gitschier:** What did you do?

**Jeffreys:** Well, I nearly had a nervous breakdown, but I kept going. It was an insane two years, 1985–1987, before the thing went commercial. We were the only lab providing any testing at all.

**Gitschier:** And then there is the local double rape/murder case in a village near Leicester. I read “The Blooding.”

**Jeffreys:** It's a good book. It's accurate.

**Gitschier:** Ah, you've answered my question. It depicted you as this chain-smoking guy in a black jumper [sweater]. What did you think about that?

**Jeffreys:** Well, let me tell you a little story.

The author was Joseph Wambaugh, an ex-LA cop who happened to read about the story in his dentist's office in *Hippocrates* magazine. He thought this is brilliant, and he took the plane straight over here and interviewed all sorts of people, myself included. My secretary had written “interview with Rambo” on my calendar. No idea who he was. And I was very cautious.

He arrived, and we did not get along terribly well, talking at cross-purposes. He wanted to dig as deep as he possibly could—that was his job as an author—and my instinct was to keep stum.

It was an extraordinary case. We were approached in 1986 by the police. They said, we've got these terrible double rape/murder cases, we have a prime suspect who has confessed to the second murder. We've heard about this DNA fingerprinting and could you use this technology, not to confirm his guilt with respect to the second murder, we know that, but to have a look at the first murder and see if we can tie him in.

So, I said we'll do this, but I explained at the outset that we wouldn't be using DNA fingerprinting, but we'd use this derived technology DNA profiling, which we thought would be much more appropriate. And we said, “Don't hold your breath. No one has ever attempted this before.”

**Gitschier:** Tell me about profiling, what it means, and why you used it instead.

**Jeffreys:** We knew that DNA fingerprints were too insensitive for forensic casework. So, we simply took out the minisatellite core probe, we went back into our libraries of DNA and cloned out the most variable single locus probes, each of which gave a simple but highly variable two-band pattern. We knew that was the way forward.

**Gitschier:** OK, back to the case.

**Jeffreys:** The forensic samples arrived, and I have to say that was a chilling moment. An ordinary academic and suddenly you've got murder samples in front of you. I remember my blood literally running cold at that point.

We put the first probe on, and the prime suspect wasn't a match [with the semen sample from the second murder]! Suddenly we were into the world of exclusion, and how many probes do you need for that? One. The result was so wacky, so totally out of keeping from what the police were expecting to see. We thought better do another one [probe]. The results were totally astonishing, totally overturned what the police had got fixed in their minds about the guilt of this prime suspect. He was released.

The police said, OK we now believe all this DNA testing, let's go and pan the entire local community and see if we can flush out the true murderer. That was all done by Home Office forensic scientists, who at that point had our DNA fingerprinting in place. But of those 5000 samples, only 500 were DNA fingerprinted. The others were all excluded by [biochemical] testing.

The upshot of that was that the true perpetrator was flushed out, and the rest is history.

**Gitschier:** Have you been in any other books?

**Jeffreys:** I've certainly turned up in all sorts of science-y books. DNA fingerprinting is now part of the curriculum for kids age 14–15 in the UK.

So, I've achieved that sort of rare status of science reaching out to the public and being understood by school kids. And literally every 2 or 3 days I get an email, mainly from the States, from school kids saying, “I've got to do a project on a famous scientist, so I've chosen you,” and I love that. I always respond.

It's great because if you think you are doing even the tiniest bit to switch people on to science, and this DNA stuff is great—OJ Simpson, the Romanovs, it's got everybody. If you can't hook people into science with that story, give up.

